# Folding Mitochondrial-Mediated Cytosolic Proteostasis Into the Mitochondrial Unfolded Protein Response

**DOI:** 10.3389/fcell.2021.715923

**Published:** 2021-09-23

**Authors:** Edmund Charles Jenkins, Mrittika Chattopadhyay, Doris Germain

**Affiliations:** Division of Hematology/Oncology, Department of Medicine, Icahn School of Medicine at Mount Sinai, Tisch Cancer Institute, New York, NY, United States

**Keywords:** proteasome, heat shock, translation, mitochondria, mitochondrial UPR, mitochondrial integrated stress response, estrogen receptor alpha

## Abstract

Several studies reported that mitochondrial stress induces cytosolic proteostasis. How mitochondrial stress activates proteostasis in the cytosol remains unclear. However, the cross-talk between the mitochondria and cytosolic proteostasis has far reaching implications for treatment of proteopathies including neurodegenerative diseases. This possibility appears within reach since selected drugs have begun to emerge as being able to stimulate mitochondrial-mediated cytosolic proteostasis. In this review, we focus on studies describing how mitochondrial stress activates proteostasis in the cytosol across multiple model organisms. A model is proposed linking mitochondrial-mediated regulation of cytosolic translation, folding capacity, ubiquitination, and proteasome degradation and autophagy as a multi layered control of cytosolic proteostasis that overlaps with the integrated stress response (ISR) and the mitochondrial unfolded protein response (UPR^mt^). By analogy to the conductor in an orchestra managing multiple instrumental sections into a dynamically integrated musical piece, the cross-talk between these signaling cascades places the mitochondria as a major conductor of cellular integrity.

## Introduction

While unique protein quality controls exist for individual organelles, disruption of the homeostasis of one organelle can affect the function of the others (Veatch et al., 2009; Hughes and Gottschling, 2012). Therefore, communication between cellular compartments is critical for the maintenance of cellular integrity.

In recent years, the communication between the mitochondria and the nucleus has gained much attention and is referred as the mitochondrial unfolded protein response (UPR^mt^). In addition, stress in both the endoplasmic reticulum and the mitochondria have been found to converge on the regulation of translation through phosphorylation and attenuation of the translation elongation factor eIF2a. This response is referred as the integrated stress response (ISR). Subsequently, a clear interconnection between the UPR^mt^ and the ISR was described. Since both of these pathways have been the topic of excellent recent reviews (Pakos-Zebrucka et al., 2016; Costa-Mattioli and Walter, 2020), they will be only discussed briefly in the current review.

The UPR^mt^ has also been linked to the regulation of cytosolic proteostasis and in addition new pathways such as the UPRam and mitochondria-to-cytosol stress response (MCSR) have been reported to regulate cytosolic proteostasis, but their relation to the ISR and the UPR^mt^ remains unclear.

**FIGURE 1:**
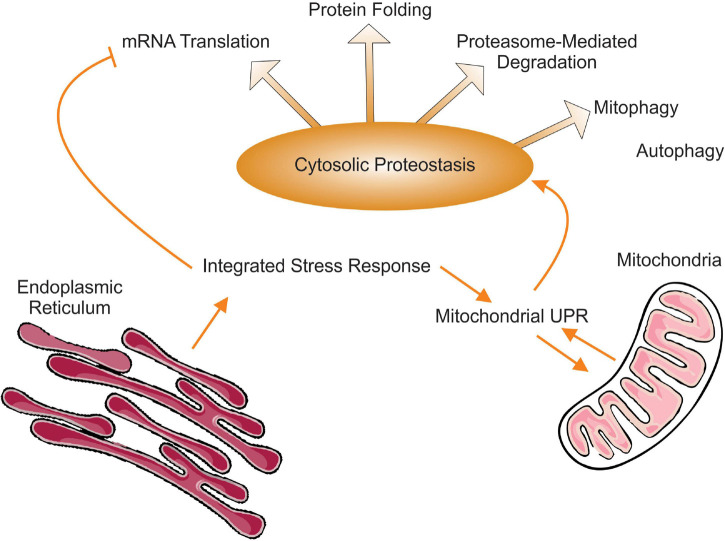
Schematic representation of the interactions between integrated stress response, the mitochondrial UPR and cytosolic proteostasis. Of note, while autophagy represents an important aspect of cytosolic proteostasis, to our knowledge mitochondrial stress and the UPR^mt^ activates mitophagy specifically but not the elimination of protein aggregates by autophagy.

Proteostasis is defined as the process by which a functional and balanced proteome is maintained. In order to be achieved, a balanced proteome requires the coordination between mRNA translation, protein folding and regulated proteasome degradation as well as autophagy (Figure 1). The picture that emerges from the studies described in this review is that in terms of proteostasis, mitochondrial stress simultaneously impacts translation, folding, proteasome-mediated degradation of proteins, and autophagy. Therefore, the ISR may in fact be even more integrated and extend well beyond the regulation of translation and the UPR^mt^ to include a well-orchestrated coordination of all steps leading to balanced and functional proteome.

### The Cross Talk Between Mitochondrial Stress and Attenuation of Cytosolic Protein Translation

The ISR refers a network of stress-activated kinases that phosphorylates the eukaryotic translation initiation factor 2 (eIF2a) leading to its inactivation and attenuation of general translation. These kinases are protein kinase R (PKR), PKR-like endoplasmic reticulum kinase (PERK), heme-regulated eIF2a kinase (HRI), and general control non-repressed kinase 2 (GCN2). An array of different stresses activates these kinases including amino acid starvation, heme deficiencies, viral infection, and stress in the endoplasmic reticulum (Wek et al., 2006; Wek and Cavener, 2007). The attenuation of translation results in a reduction in the production of newly synthesized proteins and therefore contributes to the reestablishment of proteostasis.

However, while general translation is attenuated, selected mRNAs that contain upstream open reading frames (uORFs) are selectively translated during ISR (Andreev et al., 2015). The transcription factors CCAAT/enhancer-binding protein homology protein (CHOP), the original transcription factor implicated in the mitochondrial UPR (Zhao et al., 2002), activating transcription factor 4 (ATF4) and ATF5 all contain uORF in their mRNA (Jousse et al., 2001; Vattem and Wek, 2004; Watatani et al., 2008; Palam et al., 2011). Collectively, the activation of ATF4, ATF5, and CHOP leads to increased mitochondrial proteases and chaperones, increase metabolic adaptation, and reduced oxidative phosphorylation. The roles of these transcription factors in mitochondrial biology have been extensively reviewed recently (Pakos-Zebrucka et al., 2016; McConkey, 2017; Melber and Haynes, 2018; Anderson and Haynes, 2020). However, prior to the discovery of their impact on the mitochondria, each of these transcription factors were reported to be implicated in ER stress (Zhou et al., 2008; Teske et al., 2013; Juliana et al., 2017; Yang et al., 2017). Therefore, by having distinct roles in both the mitochondria and the endoplasmic reticulum, ATF4, ATF5, and CHOP allow the reduction of stress in both organelles simultaneously (Figure 1).

In addition to CHOP, ATF4 is also directly implicated in the regulation of mitochondria proteostasis (Harding et al., 2003). Further, the Haynes lab reported that, while reactive oxygen species (ROS) are not required for the activation of ATFS-1 (the homolog of ATF5 in *C. elegans*), ROS are necessary for the GCN-2 dependent phosphorylation of eIF2a. GCN-2 is a nutrient sensor and is mainly known to be activated by starvation or amino acid depletion via deacylated tRNA (Dong et al., 2000; Zaborske et al., 2009; Perez and Kinzy, 2014). However, ROS can also stimulate GCN-2 activity by a mechanism involving the tRNA synthetase domain (Mascarenhas et al., 2008; Berlanga et al., 2010).

This suggests that these two pathways represent distinct axes of mitochondrial unfolded protein response (UPR^mt^). In agreement with this possibility, they found that deletion of GCN-2 in *C. elegans* increases the dependency on ATFS-1 for survival (Baker et al., 2012). Since the vast majority of mitochondrial proteins are translated in the cytosol, the regulation of mitochondrial chaperones and proteases by ATFS-1 and cytosolic protein synthesis by GCN-2 appear to complement each other in reducing mitochondrial proteotoxic stress.

The coordination of the ISR to mitochondrial proteostasis was further demonstrated by the finding that Tim17A, a subunit of the mitochondrial import translocase complex TIM23, which is required for the import of 99% of mitochondrial proteins, is degraded in a Yme1L dependent manner upon activation of the ISR (Chacinska et al., 2009; Schmidt et al., 2010; Rainbolt et al., 2013). The resulting decrease in protein import in the mitochondria activates the UPR^mt^ and genes implicated in mitochondrial proteostasis (Figure 1; Rainbolt et al., 2013). The stress-induced degradation of Tim17A increased stress resistance in *C. elegans* but was found to be independent of ATFS-1, indicating that additional transcription factors induce mitochondrial proteostasis genes in *C. elegans* in addition to ATFS-1 (Rainbolt et al., 2013).

### Proteostatic Stress in the Intermembrane Space of the Mitochondria Promotes the Activity of the Proteasome

While the latter study demonstrated that defect in mitochondrial proteins import due to inactivation of the Tim23 translocase complex promotes mitochondrial proteostasis, defect in mitochondrial import specifically in the inter-membrane space (IMS) of the mitochondria was shown to affect cytosolic proteostasis. Having two sub-compartments, the IMS and the matrix, importing proteins into the mitochondria requires a precise sorting of proteins destined to these respective sub-compartments. The MIA machinery is responsible for the import of proteins specifically in the IMS (Neupert and Herrmann, 2007; Chacinska et al., 2009; van der Laan et al., 2010; Endo et al., 2011). The Chacinska group performed an RNA seq analysis of a yeast strain (Mia40-1int) that is defective for import of proteins in the IMS at restrictive temperature. First, they found that inhibition of IMS proteins import leads to inhibition of protein synthesis but, unlike the ISR pathway, the reduction in proteins synthesis was due to a decrease in expression of cytosolic ribosomal proteins and proteins involved in translation (Wrobel et al., 2015). This finding indicates that diverse mitochondrial stresses result in decreased cytosolic translation, although through distinct mechanisms. Second, they found that inhibition of IMS proteins import leads to activation of the proteasome (Wrobel et al., 2015). In yeast, the coordinated expression of proteasome genes is mediated by the transcription factor Rpn4 (Kruegel et al., 2011). While, the specific role of Rpn4 was not investigated in the this study, the authors reported that the increase in proteasome activity did not correlate with an increase in the abundance of proteasome subunits. Rather, the increased activity of the proteasome was associated with increased proteasome assembly (Figure 1; Wrobel et al., 2015). The response was named the unfolded protein response activated by protein mistargeting (UPRam) and is considered to be a distinct response to the UPR^mt^, since the UPR^mt^ is best known to regulate mitochondrial proteases and chaperones.

While transcription of proteasomes subunits and assembly factors are regulated by Rpn4 in yeast, in mammals this task is mediated by Nuclear factor erythroid derived 2-related factor 1, Nrf1 also named NFE2L1 (Radhakrishnan et al., 2010; Steffen et al., 2010), which resides at the endoplasmic reticulum. Importantly Nrf1/NFE2L1 is not to be confused with the Nuclear Respiratory Factor-1, NRF-1, a transcription factor implicated in the UPR^mt^ and involved in mitochondrial biogenesis but does not regulate the proteasome. When the proteasome activity is diminished, Nrf1/NFE2L1 translocates to the nucleus due to its processing by NGLY1 and DDI2 (Radhakrishnan et al., 2010; Steffen et al., 2010; Koizumi et al., 2018) to promote the transcription of proteasome genes (for a recent review Northrop et al., 2020). Interestingly, disruption of NGLY1 affects mitochondrial function (Kong et al., 2018; Yang et al., 2018) further indicating the importance of the proteasome on mitochondrial function.

The activation of the proteasome by proteostatic stress in the IMS was also reported in a mammalian cancer cell model (Papa and Germain, 2011). The Germain group conducted this study to interrogate whether the original CHOP axis of the UPR^mt^ described by the Hoogenraad group, where overexpression of the misfolded matrix protein OTCdelta was used as a model, is also activated when misfolded proteins are in the IMS rather than the matrix. They found that proteostatic stress in the IMS does not activate CHOP and the matrix proteases and chaperones. Rather they found that stress in the IMS leads to a distinct axis of the UPR^mt^ regulated by the estrogen receptor alpha (ERα), a potent transcription factor and leads to the up-regulation of proteasome activity (Figure 1; Papa and Germain, 2011). Therefore, while stress in the endoplasmic reticulum activates the activity of the proteasome via Nrf1/NFE2L1, stress in the mitochondria activates the proteasome via the ERα. While the precise mechanism by which the ERα affects the proteasome remains to be determined, inhibition of the ERα by shRNA abolishes this effect of IMS stress on the proteasome (Papa and Germain, 2011). Considering that the ERα regulates the transcription of hundreds of genes, including transcription factors, but that the vast majority of ERα binding sites are located at a great distance from its target genes (Carroll et al., 2006), the impact of the ERα on the transcription of proteasome genes may be direct or indirect. In addition, they reported that the ERα is also necessary for the transcription of the mitochondrial biogenesis transcription factor, nuclear respiratory factor 1, NRF-1 (Papa and Germain, 2011), confirming a previous report of an estrogen receptor responsive element in the promoter of NRF-1 (Ivanova et al., 2013). Importantly and as mention above Nrf1/NFE2L1 is a transcription factor directly regulating the proteasome but not NRF-1. Mechanistically, they show that mitochondrial ROS is elevated upon accumulation of misfolded proteins in the IMS and leads to the activation of the kinase Akt, which then phosphorylates and activates the ERα (Papa and Germain, 2011). The Germain group has also shown that inhibition of the ERα does not abolish the activation of CHOP by matrix stress and conversely that inhibition of CHOP does not inhibit the activation of the ERα. Based on these findings, they concluded that the UPR^mt^ has multiple axes that regulate different cytoprotective and mito-protective outcomes (Papa and Germain, 2011).

The same group subsequently validated these findings in a disease relevant mouse model of familial ALS, where G93A-SOD1 mutant is known to accumulate in the IMS (Riar et al., 2017). This study not only validated the activation of the estrogen receptor axis of the UPR^mt^ *in vivo* and during disease progression, but also revealed significant differences in the activation of the proteasome between sexes (Riar et al., 2017). Consistent with the synergy between estrogen and Akt in the activation of the estrogen receptor, it was found that females show higher activity of proteasome than males (Riar et al., 2017).

Therefore, as with the apparent multiple mechanisms of attenuation of translation upon mitochondria stress, at least two mechanisms of proteasome activation upon mitochondria stress have been reported. However, whether these differences are conserved in divergent pathways across model systems such as yeast and *C. elegans* compared to mammalian cells remains to be clarified.

In addition to the findings that mitochondrial stress activates the proteasome, a study also reported that mitochondrial stress promotes the disassembly of the 19S regulatory lid from the 20S catalytic core of the proteasome (Livnat-Levanon et al., 2014). The fully assembled 26S proteasome promotes the degradation of poly-ubiquitinated proteins by the recognition of ubiquitin chains, followed by deubiquitination and unfolding of proteins by the19S regulatory lid, which are then pushed into the catalytic core for degradation by the chymotrypsin, trypsin-like and caspase like catalytic subunits facing the catalytic chamber. The degradation results into small peptides that are expelled into the cytoplasm (Budenholzer et al., 2017; Collins and Goldberg, 2017, for recent reviews). In absence of the 19S regulatory lid, the 20S proteasome is unable to degrade poly-ubiquitinated proteins, however, the 20S proteasome was shown to promote the degradation of unstructured proteins and oxidized proteins (Demasi and da Cunha, 2018; Kumar Deshmukh et al., 2019, for recent reviews). Treatment with antimycin A led to increase in ROS was shown to promote the dissociation of the 19S from the 20S in yeast (Livnat-Levanon et al., 2014). Poly-ubiquitinated proteins accumulated and the activity of the 20S proteasome increased following dissociation (Livnat-Levanon et al., 2014). However, this dissociation was found to be transient and the reassembly into 26S proteasome quickly restored suggesting that proteasome dissociation represents a response to acute mitochondrial stress (Livnat-Levanon et al., 2014).

Collectively, these findings suggest a potent cross-talk between mitochondrial stress and the activity of the proteasome. Based on the evidence available and if these pathways are conserved in mammalian cells, the hypothesis that emerges is that the effect of mitochondrial stress on the proteasome may fluctuate and adjust with the level of stress. In presence of acute mitochondria stress, rapid and transient dissociation and accumulation of 20S proteasome is observed, which would allow for the elimination of unstructured and oxidized proteins. Under more moderate stress conditions such as those observed by attenuation of import in the IMS or accumulation of misfolded proteins in the IMS, proteasome assembly by the UPRam and transcription of proteasome subunits by the estrogen receptor axis of the UPR^mt^ are observed. Increased 26S proteasome activity is expected to contribute to the elimination of accumulated mitochondrial precursors and accelerate the degradation of other poly-ubiquitinated proteins, therefore contributing to the rapid restoration of a balanced proteome. Clearly more studies are required to test this hypothesis as more detailed understanding of the link between mitochondrial stress and increased proteasome activity could lead to novel therapeutic intervention against proteopathies.

### Mitochondrial Stress and Cytosolic Protein Folding

The Dillin group reported the results of a screen where 12 organelles specific variants of the chaperone hsp70 were inhibited genetically and the effect of their elimination of cellular proteostasis analyzed. They found that inhibition of mitochondrial hsp70 leads to the up-regulation of cytosolic hsp60 in absence of heat shock conditions and this effect was unique to mitochondrial hsp70 as inhibition of all other 11 organelle-specific variants did not induce the same effect (Kim et al., 2016). Perhaps not surprisingly, inhibition of mitochondrial hsp70 induced the UPR^mt^ and was dependent on the transcription factors atfs-1 and dve1, but more surprisingly it also activated the heat-shock factor 1 (HSF-1), a key transcription factor for the heat shock response in the cytosol (Figure 1). This study also revealed a novel role of lipid biosynthesis in this response that was associated with decreased fatty acid oxidation and increased lipid accumulation (Kim et al., 2016). Therefore, since this pathway presented unique features and encompass both the UPR^mt^ and heat shock response (HSR), they named this response the mitochondria-to-cytosol response (MCSR) (Kim et al., 2016). Of note, the activity of the proteasome was not affected by inhibition of mitochondria hsp70 (Kim et al., 2016).

Importantly, they also tested the impact of MCSR on the toxicity of protein aggregates using a model of YFP protein fused with 35 poly-glutamine repeats and expressed in *C. elegans*. They found that activation of the MCSR reduced the accumulation and toxicity of polyQ protein aggregates in skeletal muscle and improved motility in *C. elegans* (Kim et al., 2016).

Further support to the link between heat shock response and mitochondrial stress arises from a study from the Morimoto group, who performed a screen for genes that can restore resistance to heat shock in day 2 *C. elegans* adults. This screen identified F29C4.2, which is orthologous to COX6C in human, a gene implicated in the electron transport chain (Labbadia et al., 2017). Inhibition of F29C4.2 activated the UPR^mt^ and promoted the maintenance of the heat shock response through increased binding of HSF-1 to the promoters of its target genes (Labbadia et al., 2017). No activation of the endoplasmic reticulum UPR was observed. Of note the inhibition of F29C4.2 was found to cause only mild mitochondrial stress (not acute) and resulted in increased longevity (Labbadia et al., 2017). This finding is in agreement with the notion that mitohormesis is associated with longevity (Neafsey, 1990; Rattan, 2008; Santoro et al., 2020). However, the increased longevity was not dependent on atfs-1 and the UPR^mt^ (Labbadia et al., 2017).

Further, in agreement with the Dillin group study, the toxicity of the expression of a protein containing 44 polyglutamine repeats in the intestine of *C. elegans* was reduced by inhibition of F29C4.2 (Labbadia et al., 2017).

Taken together, these studies indicate that mitochondrial stress also induce the heat shock response and cytosolic chaperones, which represents another critical layer of overall cytosolic proteostasis (Figure 1).

### The Integrated Stress Response and the Mitochondrial Unfolded Protein Response Also Impact Autophagy, Mitophagy

In addition to the chaperones and the proteasome, autophagy represents an important additional layer to maintain the cytosolic proteome. Autophagy is a well-orchestrated pathway implicating more than 30 autophagy-related (ATG) genes. Nutrient starvation was initially shown to be the mechanism of activation of autophagy (Mizushima and Komatsu, 2011). Subsequently, however, accumulation in the lumen of the endoplasmic reticulum and the UPR^ER^ was also found to activate autophagy (Deegan et al., 2013). Importantly for this review, ATF4 and CHOP, which are both implicated in the ISR, were shown to promote the transcription of several ATG genes (B’chir et al., 2013). The link between autophagy and ER stress has been recently reviewed elsewhere (Senft and Ronai, 2015).

The SIRT3 axis of the UPR^mt^ has also been reported to activate the transcription of several autophagy genes (Papa and Germain, 2014). Therefore, autophagy appears to represent yet another layer of cytosolic proteostasis that is activated by both the IRS and the UPR^mt^.

Further, link between mitochondrial dysfunction, autophagy and proteasome activity was demonstrated by the Trougakos group, who showed that decrease in proteasomal function results in severe defects in mitochondrial function (Tsakiri et al., 2019), a finding that has been reported by several independent groups using different model systems (Pellegrino and Haynes, 2015; Llanos-Gonzalez et al., 2019). The Trougakos group also reported that enhanced mitochondrial fusion and autophagy both improved the effect of proteasome dysfunction (Tsakiri et al., 2019).

Mitochondrial stress is a potent activator of mitophagy, the selective autophagy of the mitochondria. However, since this topic has been extensively covered elsewhere, this aspect will not be further discussed in the current review.

### Drugs Able to Stimulate Mitochondrial-Stress Mediated Cytosolic Proteostasis

The remarkable ability of mild mitochondrial stress to simultaneously attenuate translation, increase folding of existing proteins by induction of the heat shock response and simulate the 26S proteasome creates a unique therapeutic opportunity against proteopathies including neurodegeneration.

So far a few drugs have been identified in this setting. While their full clinical potential and precise mechanism by which they led to activation of the UPR^mt^ remains to be explored, they are nevertheless worth attention.

#### Inhibition of Mitochondrial Enzymes

Carnitine palmitoyltransferase (CPT) inhibitor perhexiline (PHX) leads to inhibition of fatty acid oxidation, CPT inhibitors are already used clinically to improve heart function. The Dillin lab showed that by inducing MCSR pathway, CPT inhibition by PHX reduces the accumulation of polyQ protein aggregates (Kim et al., 2016). Therefore, while these drugs represent potential candidates for treatment of neurodegenerative diseases associated with toxic protein aggregates, considering that perhexiline inhibits Complex IV and Complex V and moderately inhibited Complex II and Complex II and III, which cause mitochondrial dysfunction, apoptosis and hepatoxicity (Ren et al., 2020, 2021), the toxicity of these drugs is a concern.

#### Doxycycline

Doxycycline promotes the inhibition of mitochondrial translation. Treatment with doxycycline was found to activate the UPR^mt^ in *C. elegans* but not the HSR and was found to reduce amyloid beta deposits in the SH-SY5Y neuroblastoma cell line (Sorrentino et al., 2017).

Further, Doxycycline was recently shown to improve survival and reduce neuronal cell death in a mouse model of the mitochondrial disease Leigh syndrome (Perry et al., 2021). Considering that a recent clinical trial found that doxycycline did not cause major toxicity in patients (D’Souza et al., 2020), the use of doxycycline for treatment of neurodegenerative diseases appears to be feasible and safe.

In agreement with the therapeutic potential of doxycycline, the Germain group also observed sex and CNS specific regions effects of doxycycline on the proteasome and that doxycycline activates the ERα axis of UPR^mt^ in the CNS (Jenkins et al., 2021, Scientific Reports, In Press).

#### Raloxifene

There has been a long history of interest of the role of the estrogen receptor alpha (ERα) in neurodegenerative diseases especially due to the observation of sex differences that characterize these diseases and because the basis of these differences are largely unknown (Zagni et al., 2016). Several drugs have been developed to target the ERα in the context of breast cancer but, while these drugs inhibit the ERα in the breast, they were found to stimulate its activity in the CNS (Halbreich and Kahn, 2000; Littleton-Kearney et al., 2002; Miller, 2002; Veenman, 2020). This observation raised the possibility to use selective estrogen receptor modulators (SERMs) as potential therapeutic against neurodegenerative diseases. However, the initial enthusiasm of using SERMs in this context was blunted due to their failure to improve clinical outcomes in several diseases (Rapp et al., 2003; Espeland et al., 2004; Gleason et al., 2015; Henderson et al., 2015).

However, a significant oversight in the use of SERMs in these diseases is the differential effect of SERMs, including tamoxifen and raloxifene, on the transcriptional activity of the ERα (Eeckhoute et al., 2006; Lupien et al., 2008; Martinkovich et al., 2014; Jeselsohn et al., 2018) as well as the tissue specific action of the ERα (Eeckhoute et al., 2006; Lupien et al., 2008; Martinkovich et al., 2014; Jeselsohn et al., 2018). A comparative study recently reported the effects of estrogen, raloxifene, and tamoxifen in the spinal cord, a tissue affected in ALS. This study found that raloxifene specifically stimulates the ability of the ERα to promote the activity of the proteasome and delay disease progression in a mouse model of familial ALS (Jenkins et al., 2021). Importantly, the beneficial effect of raloxifene was observed in female mice but not in males (Jenkins et al., 2021). This observation indicates that mimicking the activation of the ER α axis of the UPR^mt^ leading to stimulation of proteasome activity is also a promising avenue to stimulate cytosolic proteostasis. Further, since raloxifene is widely used clinically (Maricic and Gluck, 2002; Simpson et al., 2020) and no significant toxicity is associated with this drug, the expansion of it used against neurodegeneration appears a realistic possibility.

#### Resveratrol

Resveratrol, a compound derived from red wine was shown to reduce accumulation of b-amyloid protein, an hallmark of Alzheimer’s disease. In a recent study the Wenzel group investigated the mechanism by which resveratrol mediate this effect. They found that resveratrol activates both the UPR^ER^ and the UPR^mt^ in C. elegans (Regitz et al., 2016). Further, inhibition of macro-autophagy and chaperone-mediated autophagy blocked the beneficial effect of resveratrol. Similarly, inhibition of the proteasome also blocks the effect of resveratrol (Regitz et al., 2016). However, since the beneficial effects of resveratrol are dose dependent, its clinical use against neurodegeneration remain to be determined (Jardim et al., 2018).

## Concluding Remarks

The history of the discovery of the UPR^mt^ and the ISR has been fascinating and the complexity of these pathways and the respective roles of distinct axes remain to be clearly defined. In this review, we have attempted to argue that mitochondrial stress leads to the activation of a combination of axes of these pathways that ultimately leads to a comprehensive control of cytosolic proteostasis at all levels; translation, folding and degradation by the proteasome as well as autophagy (Figure 1).

Of particular interest is the observation that proteins localized to the membrane of the endoplasmic reticulum notably PERK and NRF1/NFE2L1 contribute to the maintenance of mitochondrial function by regulating the ISR and activation of the UPR^mt^ and the direct upregulation of the proteasome, respectively. The picture that emerges is that like the UPR^mt^, the ISR may actually consists of several axes.

The fact that drugs currently used clinically begin to emerge as potential therapeutics against proteopaties by exerting moderate mitochondrial stress and activating cytosolic proteostasis represent an exciting avenue for future research. However, the success of these drugs is likely to be tissue specific. Notably, while the expression of the mitochondria import machinery is ubiquitous (Bauer et al., 1999), it was noted that the sensitivities to stress-regulated translation attenuation is tissue specific, and also indicated that the regulation of TIM23 may vary between tissues (Rainbolt et al., 2013). Similarly, the level of expression of chaperones and the overall proteome are also highly tissue specific and can affect each other through transcellular chaperone signaling (van Oosten-Hawle et al., 2013; van Oosten-Hawle and Morimoto, 2014). A recent report indicated that the activity of the proteasome is tissue and sex -specific (Jenkins et al., 2020) supporting the notion of a wide number of different species of proteasomes (Dahlmann, 2016). Combined with the fact that the number of individual mitochondrion, as well as the wide variation in the shape and distribution of the mitochondrial network between tissues, it appears very important to apply nuanced interpretation of the results obtained in future investigations of the cross talk between the mitochondria and cytosolic proteostasis, thus allowing for the complexity that results from differences between sexes and tissues.

## Author Contributions

DG and EJ made the figure. All authors have participated in the writing of this review.

## Conflict of Interest

The authors declare that the research was conducted in the absence of any commercial or financial relationships that could be construed as a potential conflict of interest.

## Publisher’s Note

All claims expressed in this article are solely those of the authors and do not necessarily represent those of their affiliated organizations, or those of the publisher, the editors and the reviewers. Any product that may be evaluated in this article, or claim that may be made by its manufacturer, is not guaranteed or endorsed by the publisher.
